# Evaluation of the Collaborative Use of an Evidence-Based Care Bundle in Emergency Laparotomy

**DOI:** 10.1001/jamasurg.2019.0145

**Published:** 2019-03-20

**Authors:** Geeta Aggarwal, Carol J. Peden, Mohammed A. Mohammed, Anne Pullyblank, Ben Williams, Timothy Stephens, Suzanne Kellett, James Kirkby-Bott, Nial Quiney

**Affiliations:** 1 Department of Anesthesiology, Royal Surrey County Hospital, Guildford, United Kingdom; 2 Department of Anesthesiology, Keck School of Medicine, University of Southern California, Los Angeles; 3 Faculty of Health Studies, University of Bradford, Bradford, United Kingdom; 4 Department of Surgery, North Bristol Hospital, Bristol, United Kingdom; 5 West of England Academic Health Science Network, Bristol, United Kingdom; 6 Kent Surrey Sussex Academic Health Science Network, Crawley, United Kingdom; 7 Critical Care and Perioperative Medicine Research Group, William Harvey Research Institute, Queen Mary University of London, London, United Kingdom; 8 Department of Anesthesiology, University Hospital Southampton, Southampton, United Kingdom; 9 Department of Surgery, University Hospital Southampton, Southampton, United Kingdom

## Abstract

**Importance:**

Patients undergoing emergency laparotomy have high mortality, but few studies exist to improve outcomes for these patients.

**Objective:**

To assess whether a collaborative approach to implement a 6-point care bundle is associated with reduction in mortality and length of stay and improvement in the delivery of standards of care across a group of hospitals.

**Design, Setting, and Participants:**

The Emergency Laparotomy Collaborative (ELC) was a UK-based prospective quality improvement study of the implementation of a care bundle provided to patients requiring emergency laparotomy between October 1, 2015, and September 30, 2017. Participants were 28 National Health Service hospitals and emergency surgical patients who were treated at these hospitals and whose data were entered into the National Emergency Laparotomy Audit (NELA) database. Post-ELC implementation outcomes were compared with baseline data from July 1, 2014, to September 30, 2015. Data entry and collection were performed through the NELA.

**Interventions:**

A 6-point, evidence-based care bundle was used. The bundle included prompt measurement of blood lactate levels, early review and treatment for sepsis, transfer to the operating room within defined time goals after the decision to operate, use of goal-directed fluid therapy, postoperative admission to an intensive care unit, and multidisciplinary involvement of senior clinicians in the decision and delivery of perioperative care. Change management and leadership coaching were provided to ELC leadership teams.

**Main Outcome and Measures:**

Primary outcomes were in-hospital mortality, both crude and Portsmouth Physiological and Operative Severity Score for the enumeration of Mortality and morbidity (P-POSSUM) risk-adjusted, and length of stay. Secondary outcomes were the changes after implementation of the separate metrics in the care bundle.

**Results:**

A total of 28 hospitals participated in the ELC and completed the project. The baseline group included 5562 patients (2937 female [52.8%] and a mean [range] age of 65.3 [18.0-114.0] years), whereas the post-ELC group had 9247 patients (4911 female [53.1%] and a mean [range] age of 65.0 [18.0-99.0] years). Unadjusted mortality rate decreased from 9.8% at baseline to 8.3% in year 2 of the project, and so did risk-adjusted mortality from a baseline of 5.3% to 4.5% post-ELC. Mean length of stay decreased from 20.1 days during year 1 to 18.9 days during year 2. Significant changes in 5 of the 6 metrics in the care bundle were achieved.

**Conclusions and Relevance:**

A collaborative approach using a quality improvement methodology and a care bundle appeared to be effective in reducing mortality and length of stay in emergency laparotomy, suggesting that hospitals should adopt such an approach to see better patient outcomes and care delivery performance.

## Key Points

**Question:**

Is a quality improvement collaborative approach to implementation of a care bundle associated with reductions in mortality from emergency laparotomy?

**Findings:**

In this study of a collaborative project involving 28 hospitals and a total of 14 809 patients, reductions in mortality and length of stay were seen after implementation of a care bundle. Improvement took time to occur and was not seen until the second year of the collaborative project.

**Meaning:**

The findings suggest that hospitals should consider adopting a care bundle approach and participating in a collaborative group to see improvement in outcomes for patients undergoing emergency laparotomy.

## Teaser

This quality improvement study evaluates mortality and other outcomes data of patients who underwent emergency laparotomy before and after they received collaborative care.

## Introduction

Emergency general surgery occurs commonly,^1^ and patients undergoing major nontrauma nonvascular intra-abdominal operation or emergency laparotomy form a specific subset of emergency general surgical patients. Mortality and morbidity rates are high for patients undergoing emergency laparotomy, with reports from the United Kingdom,^2,3,4^ United States,^5^ and Denmark^6^ suggesting a 30-day mortality of between 10% and 18%. In a UK study^3^ carried out over 3 months, crude 30-day mortality for emergency laparotomy across 27 hospitals varied between 3% and 45%. These mortality figures are substantially higher than the mortality rates for elective surgical procedures for which in-hospital mortality rates of 1% to 2% are usually reported for even the most complex procedures.^7^ To date, few studies exist to improve outcomes for patients requiring emergency laparotomy.

Underlying the observed wide variation in mortality are considerable differences in the patients undergoing emergency laparotomy and in the delivery of their care.^8^ Although it may be difficult to control for patient variation at presentation, evidence highlights the wide variation in delivering key aspects of care.^4,8,9,10,11^ This variation includes inconsistencies in initiating prompt patient resuscitation, management of common acute physiologic changes,^12^ communication between professionals, understanding of patient risk,^8^ use of perioperative goal-directed fluid resuscitation,^3^ admission of patients after a surgical procedure to the intensive care unit,^5^ and involvement by senior surgeons and anesthesiologists in the care of patients.^2^

In the United Kingdom, the Royal College of Surgeons of England attempted to define standards of care that should be considered for the management of patients undergoing emergency laparotomy.^13^ In addition, the national Healthcare Quality Improvement Partnership funded a mandatory audit, the National Emergency Laparotomy Audit (NELA), to record the delivery of key process measures and outcomes for all patients in England and Wales who undergo emergency laparotomy.^2^

With the aim of reducing mortality for emergency laparotomy, a group of 4 hospitals in England used a care bundle approach to implement the standards of care recommended for the higher-risk surgical patient.^13^ The results showed a 25% reduction in crude 30-day mortality and a 42% reduction in the Portsmouth Physiological and Operative Severity Score for the enumeration of Mortality and morbidity (P-POSSUM)^14^ risk-adjusted mortality at 30 days.^15^ Supporting these improvements in outcome were similar substantial improvements in delivery in many key processes of care. Two further studies^16,17^ from Denmark used a similar approach with more than 700 patients and showed a similar 25% reduction in crude hospital mortality.

The 3 studies^15,16,17^ used a number of evidence-based standards of care that, when consistently delivered, brought about substantial improvements in patient outcomes. These standards of care include the (1) use of an early warning score^18^ or blood lactate level measurement to aid immediate resuscitation and escalation; (2) early identification of sepsis and early administration of broad-spectrum antibiotics, as recommended by the Surviving Sepsis Campaign^19^; (3) early transfer to the operating room (OR) to carry out definitive surgical treatment and drainage and removal of septic material^19^; (4) use of perioperative goal-directed fluid therapy (GDFT) to guide fluid resuscitation^20^; (5) admission of all patients to the intensive care unit after a surgical procedure^9,21^; and (6) involvement of senior clinicians in the decision to proceed to surgical treatment and throughout the surgical procedure.^13^

The aim of this study was to assess whether a quality improvement (QI) collaborative approach to implement a care bundle for patients undergoing emergency laparotomy across a large hospital group could be associated with a reduction in unadjusted and P-POSSUM risk-adjusted in-hospital mortality capped at 30 days, reduction in inpatient length of stay (LOS), and improvement in the delivery of agreed-on quality standards of care.

## Methods

This study was a QI project in the United Kingdom, called the Emergency Laparotomy Collaborative (ELC), involving 28 National Health Service hospitals with inpatient bed capacity between 246 and 1300. The ELC design was based on an Institute for Healthcare Improvement Breakthrough Series collaborative approach^22^ of hospital teams meeting every 3 months. Between these meetings, the teams were supported by improvement teams from 3 local Academic Health Science Network groups.^23^ The care bundle implemented is shown in the Box. An assessment of the study was completed to determine its alignment with national guidance,^24^ which confirmed the project fell outside the area of research and required no further ethical approvals or informed consent. Data from the NELA were collected by each participating ELC hospital with national ethical approval for that data set. Each hospital was asked to register its participation in the project with its own research and development panel.

Box. **How to Save Lives in Emergency Laparotomy**

Screen patient
  NEWS/SIRS/arterial lactate level
Assess whether patient has signs of sepsis
  Treat with antibiotics within 1 h
Move patient to operating room
  Move to operating room within 6 h of decision to operate
Consultant surgeon and anesthesiologist
  In operating room
Monitor cardiac output
  Goal-directed fluid therapy
ICU for all patients

Abbreviations: ICU, intensive care unit; NEWS, National Early Warning Score; SIRS, Systemic Inflammatory Response Syndrome.

Adapted from the Emergency Laparotomy Collaborative.

The hospitals were located across the south of England. All consecutive patients who underwent emergency laparotomy were included. Patients were followed up for a maximum of 30 days after the surgical procedure or until discharge or death if this occurred before 30 days. No patient selection or grouping was carried out other than using the inclusion and exclusion criteria as identified in the NELA data set during the study period.^2^

A multidisciplinary local implementation group was formed in each hospital, and the group included general surgeons, anesthesiologists, intensivists, nurses, and QI specialists. Hospitals submitted their anonymized NELA data set for the 15 months preceding the start of the project to act as their own baseline. After the launch of the project on October 1, 2015, hospitals submitted their ongoing anonymized NELA data to a central database every 3 months for the following 24 months of the project.

The ELC project had a leadership board composed of clinicians, QI experts, data analysts, and program managers. This group met regularly throughout the life of the project. The 24-month program of QI included clinical evidence review, QI methodology, leadership and negotiation coaching, promotion of collaborative learning and sharing of new ideas, and sustainability development.

The model for improvement^25^ was used to coach teams on the plan-do-study-act cycles. This teaching was combined with other elements such as systems analysis, driver diagrams, and performance monitoring using time series data. To help hospitals own their real-time data, teaching on data use and analysis was provided. Coaching was also provided to assist teams to promote behavioral change.^26^ The second 12-month period focused on leadership and negotiation skills.

Data on adherence to the 6-point care bundle were prospectively collected for each patient undergoing emergency laparotomy. Aggregate quarterly performance data for each hospital were shared across the collaborative group in the form of run charts and a comparative dashboard.

The primary outcomes were in-hospital (truncated at 30 days) mortality, both crude and P-POSSUM risk-adjusted, and LOS. The secondary outcomes were the changes after implementation of the separate metrics in the care bundle.

Baseline data were collected from July 1, 2014, to September 30, 2015 (months 1 to 15), and prospective (post-ELC implementation) data were obtained from October 1, 2015, to September 30, 2017 (months 16 to 39).

### Statistical Analysis

Initially, the statistical significance of changes, pre-ELC compared with post-ELC, in continuous variables (age, blood lactate level, systolic blood pressure, serum creatinine level, Glasgow Coma Scale score [score range: 1-15, with the highest score indicating complete consciousness], number of patients per month) was assessed using linear regression models. Likewise, quantile (P-POSSUM risk), logistic (male and type of operation), and ordinal (American Society of Anaesthesiologists physical status grade) regression models were used for other variables. Two-sided *P* values were obtained from specific statistical models, for which *P* < .05 was statistically significant.

The 10 primary and secondary outcomes (the quality indicators) were assessed for evidence of improvement using Shewhart statistical process control charts. The statistical process control methodology is a branch of statistical tool that combines rigorous time series analysis with graphical presentation of data.^27^ This technique is particularly useful in the context of real-world large-scale change in which the control of independent variables is not always possible, in the way it is in more traditional experimental approaches.^28^ Statistical process control is increasingly being recognized as the optimal way of assessing QI projects in health care.^29,30,31^

Monthly arithmetic means for each of the 10 quality indicators were plotted on time series charts. A baseline was constructed for the first 15 data points (from June 2014 through September 2015), and ongoing data were plotted on a monthly basis. For each of these charts, the expected mean value and upper and lower control limits were plotted (set at 3 SDs from the mean); these control limits are not CIs and cannot be interpreted in the same way. The charts were then inspected for common cause variation (random fluctuation) and special cause variation (changes due to external factors). Special cause variation or a substantial change not due to natural variation was identified, either when the mean monthly performance broached the upper or lower control limits or when 8 consecutive months of performance lay on 1 side of the mean line. The software used for the statistical analysis was SQCpack, version 7 (PQ Systems). Special cause variation was taken as a clinically and statistically significant change.

## Results

A total of 28 hospitals participated in the ELC and completed the project. Aggregate-level patient demographics are shown in the Table. The baseline group included 5562 patients (2937 female [52.8%] and a mean [range] age of 65.3 [18-114] years), whereas the post-ELC group had 9247 patients (4911 female [53.1%] and a mean [range] age of 65 [18-99] years). No difference in age and sex was found. No significant difference was identified in median (interquartile range [IQR]) P-POSSUM (7.00% [2.7% to 21.9%] vs 6.30% [2.5% to 19.4%]; *P* = .002) and American Society of Anaesthesiologists physical status grades. No differences were identified in preoperative median (IQR) blood lactate level (1.4 [0-20] mmol/L vs 1.4 [1-20] mmol/L; *P* > .99) (to convert to milligrams per deciliter, multiply by 0.111) and other physiologic variables between the control and post-ELC groups. The most common surgical procedure type is contained in the eFigure in the Supplement.

**Table.** Comparison Between Baseline Group and Post–Emergency Laparotomy Collaborative Implementation Group

Variable	Baseline Group, mo 1-15 (n = 5562)	Post-ELC Implementation Group, mo 16-39 (n = 9247)	*P* Value
Age, mean (range), y	65.3 (18-114)	65 (18-99)	.33
Sex, No. (%)			
Male	2625 (47.2)	4336 (46.9)	.72
Female	2937 (52.8)	4911 (53.1)	
No. of patients per mo, mean	371.6	386.9	.13
P-POSSUM, No. (%)			
Median (IQR)	7.00 (2.7-21.9)	6.30 (2.5-19.4)	.002
0-10.0	3233 (58.1)	5661 (61.2)	
10.1-20.0	834 (15.0)	1311 (14.2)	
20.1-30.0	387 (7.0)	670 (7.2)	
30.1-40.0	279 (5.0)	419 (4.5)	
40.1-50.0	196 (3.5)	286 (3.1)	
50.1-60.0	147 (2.6)	232 (2.5)	
60.1-70.0	142 (2.6)	210 (2.3)	
70.1-80.0	119 (2.1)	167 (1.8)	
80.1-90.0	116 (2.1)	150 (1.6)	
90.1-100	109 (2.0)	141 (1.5)	
ASA grade, No. (%)			
1	554 (9.96)	1017 (11.00)	.005
2	1988 (35.74)	3358 (36.31)	
3	1976 (35.53)	3294 (35.62	
4	936 (16.83)	1449 (15.67)	
5	108 (1.94)	129 (1.40)	
Preoperative physiologic variables			
Blood lactate, median (range), mmol/L	1.4 (0-20)	1.4 (0.1-20)	>.99
Systolic blood pressure, mean (range), mm Hg	126.9 (12-226)	127.1 (10.6-225)	.63
Glasgow Coma Scale score,^a^ mean (range)	14.7 (3-15)	14.7 (3-15)	.47
Serum creatinine, mean (range), μmol/L	93.2 (0.8-1200)	91.1 (0.2-1083)	.06

Abbreviations: ASA, American Society of Anaesthesiologists physical status; ELC, Emergency Laparotomy Collaborative; IQR, interquartile range; P-POSSUM, Portsmouth Physiological and Operative Severity Score for the enumeration of Mortality and morbidity. SI conversion factors: To convert lactate to milligrams per deciliter, multiply by 0.111; creatinine to milligrams per deciliter, multiply by 88.4.

^a^ Glasgow Coma Scale score range: 1-15, with the highest score indicating complete consciousness.

**Figure 1.**
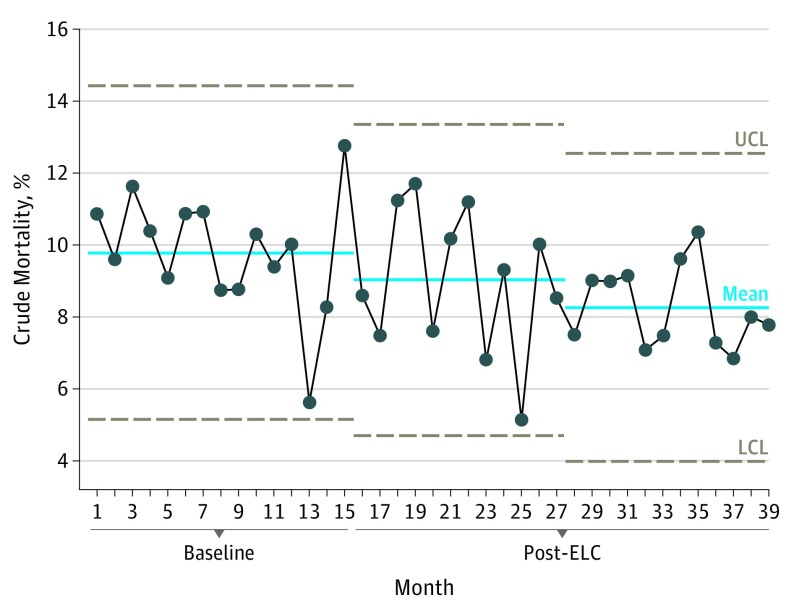
Change in Crude Mortality This statistical process control chart shows the stepwise reductions in 30-day unadjusted crude mortality. Months 1 to 15 depict the baseline data (ie, no intervention or care bundle from the Emergency Laparotomy Collaborative [ELC]); post-ELC months 16 to 27, year 1 change; and post-ELC months 28 to 39, year 2 change. LCL indicates lower control limit; UCL, upper control limit.

During the ELC implementation period, a significant reduction was observed in both crude and P-POSSUM risk-adjusted mortality. Unadjusted mortality rate was 9.8% in the baseline period, fell to 9.0% in months 15 to 27, and declined again to 8.3% in months 28 to 39 (Figure 1). A significant change in mortality was observed after month 27.

The P-POSSUM risk-adjusted mortality also fell during the study period from 5.5% at baseline to 5.1% in months 15 to 27 and 4.5% in months 28 to 39. Again, a significant change was identified after month 27.

**Figure 2.**
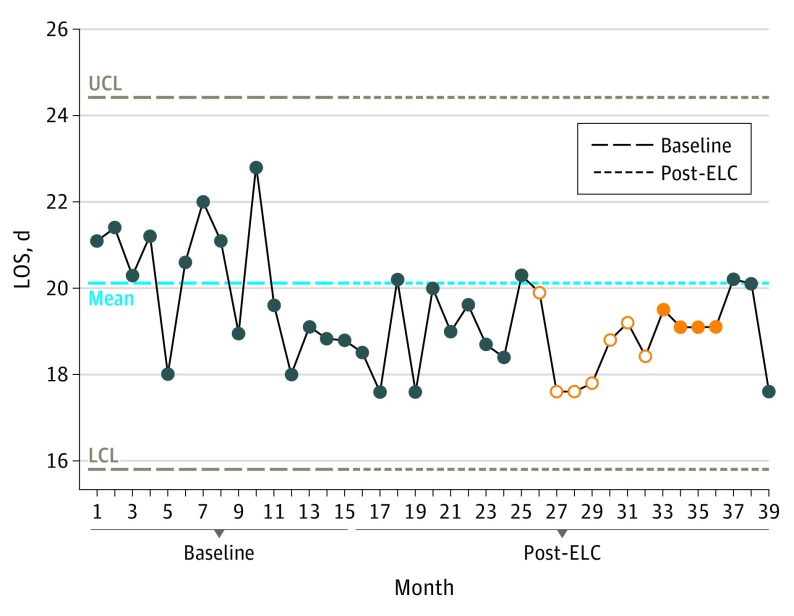
Change in Length of Stay (LOS) This statistical process control chart shows the change in baseline LOS. The mean baseline LOS was 20.1 days, which decreased to 18.9 days in post–ELC (Emergency Laparotomy Collaborative) months 13 to 27 and remained at 18.9 days for post-ELC months 28 to 39. The dark blue circles are monthly data readings; the filled orange circles are significant changes on 1 side of the mean line, indicating significance; and the empty orange circles are data points that lead up to significance. If more than 8 points lie on 1 side of the mean line, then the change is significant, which includes empty orange circles and filled orange circles. If the points cross the upper control limit (UCL) or the lower control limit (LCL), this is highly significant.

The baseline LOS mean was 20.1 days, which decreased to 18.9 days during year 1 and remained at 18.9 days during year 2 of ELC implementation. A significant change in patient LOS occurred between months 26 to 36, but this change was not sustained beyond month 36 (Figure 2).

A significant change in the P-POSSUM was identified during the study period. Overall, the preoperative P-POSSUM risk of death in the control group was 17.7%, which was reduced to 16.6% in months 16 to 27 and 15.5% in months 28 to 39 of the ELC implementation. The preoperative P-POSSUM showed a significant reduction from month 20 onward, and this decrease was sustained throughout the project.

**Figure 3.**
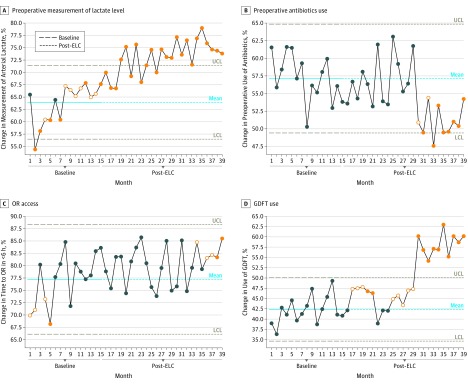
Baseline to Post–Emergency Laparotomy Collaborative (ELC) Changes by Lactate Level, Antibiotics Use, Operating Room (OR) Access, and Goal-Directed Fluid Therapy (GDFT) Use A, Changes in the measurement of blood lactate level from baseline (63.9%) to post-ELC implementation year 1 (71.2%) and year 2 (74.9%), a significant change that crossed the upper control limit (UCL) of the statistical process chart. B, Changes in the use of antibiotics before OR arrival: 57.1% of patients received antibiotics during baseline, which decreased to 56.6% in year 1 and 52.3% in year 2. C, Changes in the percentage of patients who entered the OR within 6 hours of booking, which was 77.2% at baseline but increased to 79.4% in months 16 to 27 and to 80.8% in year 2. D, Changes in the use of GDFT, which was less than 42.3% preoperatively but increased beginning in month 25 onward, a significant change that was sustained and crossed the UCL. The dark blue circles are monthly data readings; the filled orange circles are significant changes on 1 side of the mean line, indicating significance; and the empty orange circles are data points that lead up to significance. If more than 8 points lie on 1 side of the mean line, then the change is significant, which includes empty orange circles and filled orange circles. If the points cross the UCL or the lower control limit (LCL), this is highly significant.

**Figure 4.**
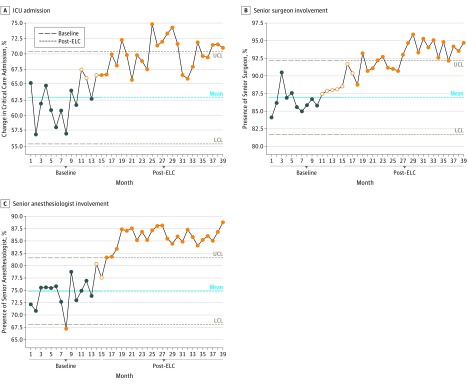
Baseline to Post–Emergency Laparotomy Collaborative (ELC) Changes by Intensive Care Unit (ICU) Admission and Surgeon and Anesthesiologist Involvement Changes in the admission rate to the ICU just before ELC implementation (month 14), which was a significant and sustained change that crossed the upper control limit (UCL) (A); the direct involvement of a senior surgeon, which occurred after month 18, crossed the UCL, and was significant (B); and the direct involvement of a senior anesthesiologist experienced by 74.8% of patients at baseline and increased to 85.8% in months 16 to 27 and was sustained in months 28 to 39 (C). See the caption to Figure 3 for explanation of dark blue circles, filled orange circles, and the empty orange circles. LCL indicates lower control limit.

Aggregate-level data for all hospital metrics are shown in Figure 3 and Figure 4. In the baseline period, 63.9% of patients (3554 of 5562) had their blood lactate levels measured before arrival in the OR. This percentage increased to 71.2% (3381 patients) in months 16 to 27 and to 74.9% (3372 patients) in months 28 to 39. A significant improvement was identified that started before the beginning of the ELC implementation and was sustained and increased throughout the ELC project (Figure 3A).

In the baseline period, 2875 (57.1%) of 5562 patients had antibiotics administered before arrival in the OR, and this number reduced to 2688 (56.6%) of 4748 patients in months 16 to 27 and to 2354 (52.3%) of 4499 patients in months 28 to 39. A significant deterioration was identified that started at month 30 and continued until the end of the project (Figure 3B).

The percentage of patients who entered the OR within 6 hours of booking was 77.2% in the baseline period. In months 16 to 27, this percentage increased to 79.4% and then to 80.8% in months 28 to 39. Overall, the improvement in access to the OR that started just before the start of the ELC project lasted from month 1 to month 3. This improvement was not sustained throughout the implementation, but it occurred again from months 34 to 39 (Figure 3C).

The use of GDFT in the OR is shown in Figure 3D. Before the start of ELC, 42.3% of patients (2353 of 5562) were managed using GDFT. This percentage increased to 44.5% (2115 of 4748 patients) in months 16 to 27 and again to 56.3% (2534 of 4499 patients) in months 28 to 39. A significant change in the use of GDFT occurred from month 25 and was sustained.

The admission rate to the intensive care unit before the ELC project was 62.9%. Again, a significant change in admission rate was seen starting just before the ELC implementation (month 14) and continued to improve throughout the project. The data show not only a significant but also a sustained change (Figure 4E).

Direct involvement by a senior surgeon was experienced by 87.0% of patients (4839 of 5562) before the ELC implementation. During months 16 to 27, this involvement increased to 91.4% (4340 of 4748 patients) and to 94.2% (4238 of 4499 patients) during months 28 to 39. The improvements in compliance with this metric started before the project (month 11) but continued throughout the implementation. A significant change occurred after month 18 (Figure 4B).

Before the ELC project, 74.8% of patients (4160 of 5562) experienced the direct involvement of a senior anesthesiologist. A significant change was seen, increasing involvement to 85.8% (4075 of 4748 patients) in months 16 to 27, which was sustained in months 28 to 39 (Figure 4C).

## Discussion

This study showed a reduction in unadjusted mortality rate and LOS as well as changes in many of the care bundle metrics after ELC implementation, suggesting that improvements in the delivery of care can be achieved. Metrics were seen to change at different rates. More marked changes occurred in the second year of the project, supporting the concept that improvement work takes time to establish.^32^ Better attendance by senior clinicians occurred early, as did the measurement of blood lactate levels and admission to the intensive care unit. Improvement in accessing the OR was often not maintained, and sustained change occurred late in the project, suggesting that this target was more complex and may first require substantial upgrades to the system at many levels. Better use of GDFT was significant but did not occur until month 17.

The use of antibiotics declined during the project, especially during the later stages of ELC implementation. This finding is surprising in view of the concurrent focus in the United Kingdom to improve identification and early treatment of patients with sepsis. One explanation may be the observed change in case mix, with fewer other cases included in the database in the implementation period than in the baseline period. Another explanation could be that the NELA data set did not allow us to distinguish patients who showed signs of sepsis and required early antibiotics from patients who were not in septic shock.

Considerably more other procedures were performed in the baseline group compared with the intervention (or post-ELC) group (eFigure in the Supplement). The use of the NELA database was relatively new at the start of the baseline period, and clinicians were likely not completely familiar with the specific codes used by the NELA database.

A reduction in the median P-POSSUM risk-adjusted mortality at 30 days was identified, and several possibilities may account for this decrease. Patient selection might have changed, but overall patient accrual rate and physiologic variables remained unchanged. Patients with high P-POSSUM risk-adjusted mortality at 30 days may have been denied for a surgical procedure, but again no evidence supports this possibility when looking at the P-POSSUM distribution. The care bundle itself may have advantages for the recorded P-POSSUM. The measurement of blood lactate level or the recording of the early warning score may have prompted earlier patient resuscitation, associated with improved physiologic variables and reduced overall P-POSSUM.

The ELC project had several features to encourage success. The care bundle approach offered a small number of simple, evidence-based metrics on which teams could focus their QI work, and collaboration among a number of hospitals has been shown to be more effective than hospitals working alone on improvement projects.^33,34^ The Michigan Surgical Quality Collaborative has demonstrated this very effectively.^35^ The use of frequent and timely data feedback has been shown to be a good indicator of successful QI initiatives.^36^ Highlighting and providing data to clinicians in an accessible manner to show their performance against peers, as was done using our dashboard, has also been shown to improve performance.^37^

### Strengths and Limitations

The study has strengths, including its use of established improvement science methodology, the size of the collaborative group, and the large cohort of patients. This study also has several limitations. The NELA data set for data entry and baseline data was not designed specifically for the care bundle metrics. Another limitation is that the project took place against a backdrop of national interest in improving outcomes for emergency laparotomy. Distinguishing improvements owing to the ELC project from those associated with the prevailing trend is challenging. In addition, the availability of data for the treatment of sepsis was not ideal. Identifying those patients who should have received antibiotics when indicated would have been more useful.

## Conclusions

The 28 participating hospitals in this collaborative project used a QI methodology and a care bundle and appeared to have substantial gains in both mortality rate and LOS. Significant improvements in recognized quality standards of care were achieved. Hospitals wishing for better outcomes for patients requiring emergency laparotomy should consider adopting a care bundle approach and participating in a QI collaborative group to see improvement in performance and reduction in mortality.

## Notes

Supplement. **eFigure.** Bar Chart Showing the Commonest Procedures Under the Umbrella Term “Emergency Laparotomy” Carried Out During Baseline and Intervention
