# Supplementary material for: Evaluation of the Collaborative Use of an Evidence-Based Care Bundle in Emergency Laparotomy
Source: JAMA Surg. 2019 Mar 20;154(5):e190145. doi: 10.1001/jamasurg.2019.0145 (PMC6537778; doi:10.1001/jamasurg.2019.0145)
Supplement: Supplement. — eFigure. Bar Chart Showing the Commonest Procedures Under the Umbrella Term “Emergency Laparotomy” Carried Out During Baseline and Intervention [file jamasurg-154-e190145-s001.pdf]

Supplementary Online Content

Aggarwal G, Peden CJ, Mohammed MA, et al; Emergency Laparotomy Collaborative. Evaluation of the collaborative use of an evidence-based care bundle in emergency laparotomy. *JAMA Surg.* Published online March 20, 2019. doi:10.1001/jamasurg.2019.0145

eFigure. Bar Chart Showing the Commonest Procedures Under the Umbrella Term "Emergency Laparotomy" Carried Out During Baseline and Intervention

This supplementary material has been provided by the authors to give readers additional information about their work.

© 2019 Aggarwal G et al. *JAMA Surgery*.

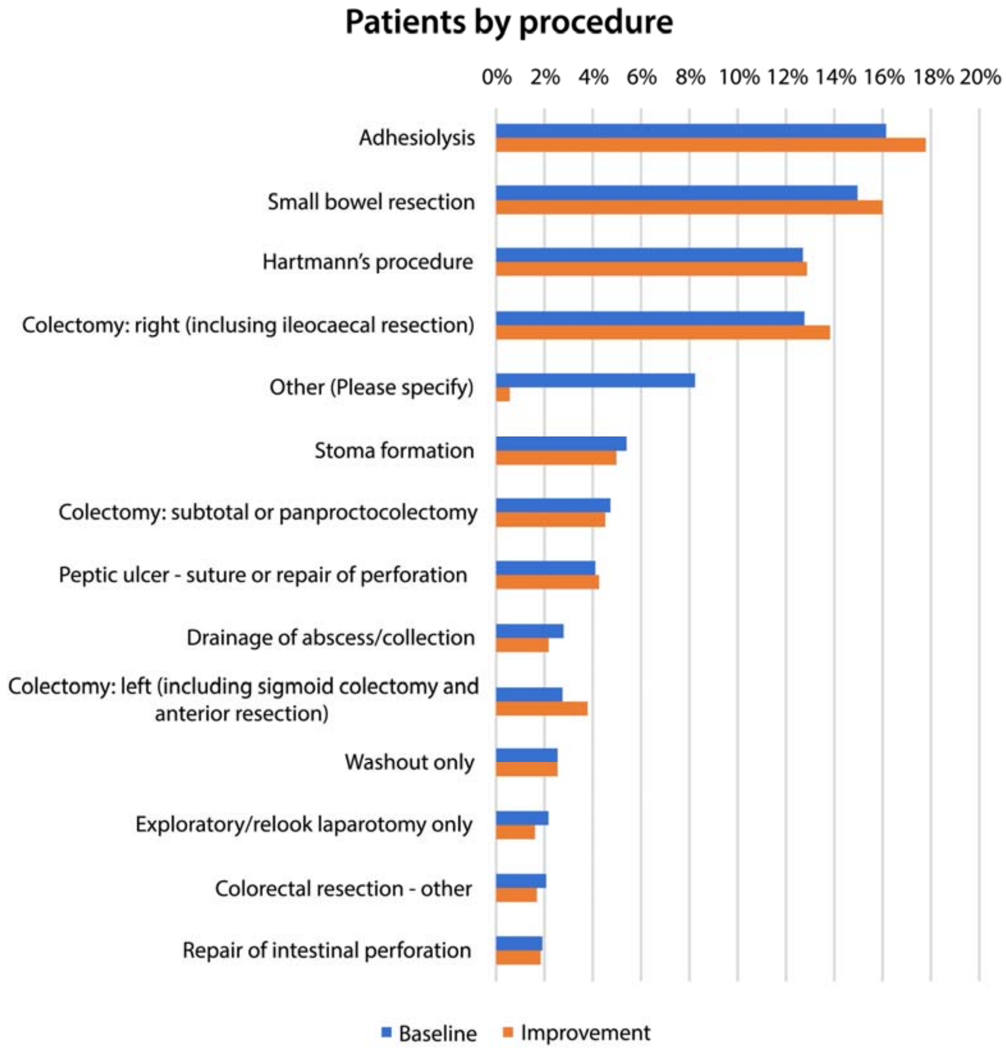

© 2019 Aggarwal G et al. *JAMA Surgery*.
